# Comparing the Effectiveness, Tolerability, and Acceptability of Heated Tobacco Products and Refillable Electronic Cigarettes for Cigarette Substitution (CEASEFIRE): Randomized Controlled Trial

**DOI:** 10.2196/42628

**Published:** 2023-04-04

**Authors:** Pasquale Caponnetto, Davide Campagna, Marilena Maglia, Francesca Benfatto, Rosalia Emma, Massimo Caruso, Grazia Caci, Barbara Busà, Alfio Pennisi, Maurizio Ceracchi, Marcello Migliore, Maria Signorelli

**Affiliations:** 1 Center of Excellence for the Acceleration of Harm Reduction University of Catania Catania Italy; 2 Department of Educational Sciences, University of Catania Catania Italy; 3 Eclat Spin off srl University of Catania Catania Italy; 4 Department of Clinical & Experimental Medicine University of Catania Catania Italy; 5 Unità Operativa Complessa Medicina Accettazione Urgenza, University Teaching Hospital University of Catania Catania Italy; 6 Department of Biomedical and Biotechnological Sciences University of Catania Catania Italy; 7 Unit of Infectious Diseases Department of Clinical and Experimental Medicine University of Messina Messina Italy; 8 Dipartimento Emergenza-Urgenza Farmacia presidio ospedaliero centro Azienda Ospedaliera di Rilievo Nazionale e di Alta Specializzazione Garibaldi Catania Italy; 9 Department of Respiratory Medicine Hospital Clinics "Musumeci-Gecas" Catania Italy; 10 Fullcro Srl Roma Italy; 11 Department of Surgery and Medical Specialties University of Catania Catania Italy; 12 Minimally Invasive Thoracic Surgery and New Technology University Hospital of Catania Catania Italy; 13 Department of Clinical and Experimental Medicine Psychiatry Unit University of Catania Catania Italy

**Keywords:** harm reduction, heat not burn, electronic cigarettes, smoking cessation, smoking reduction, e-cigarette, public health, psychological well-being

## Abstract

**Background:**

People who smoke and who face challenges trying to quit or wish to continue to smoke may benefit by switching from traditional cigarettes to noncombustible nicotine delivery alternatives, such as heated tobacco products (HTPs) and electronic cigarettes (ECs). HTPs and ECs are being increasingly used to quit smoking, but there are limited data about their effectiveness.

**Objective:**

We conducted the first randomized controlled trial comparing quit rates between HTPs and ECs among people who smoke and do not intend to quit.

**Methods:**

We conducted a 12-week randomized noninferiority switching trial to compare effectiveness, tolerability, and product satisfaction between HTPs (IQOS 2.4 Plus) and refillable ECs (JustFog Q16) among people who do not intend to quit. The cessation intervention included motivational counseling. The primary endpoint of the study was the carbon monoxide–confirmed continuous abstinence rate from week 4 to week 12 (CAR weeks 4-12). The secondary endpoints included the continuous self-reported ≥50% reduction in cigarette consumption rate (continuous reduction rate) from week 4 to week 12 (CRR weeks 4-12) and 7-day point prevalence of smoking abstinence.

**Results:**

A total of 211 participants completed the study. High quit rates (CAR weeks 4-12) of 39.1% (43/110) and 30.8% (33/107) were observed for IQOS-HTP and JustFog-EC, respectively. The between-group difference for the CAR weeks 4-12 was not significant (*P*=.20). The CRR weeks 4-12 values for IQOS-HTP and JustFog-EC were 46.4% (51/110) and 39.3% (42/107), respectively, and the between-group difference was not significant (*P*=.24). At week 12, the 7-day point prevalence of smoking abstinence values for IQOS-HTP and JustFog-EC were 54.5% (60/110) and 41.1% (44/107), respectively. The most frequent adverse events were cough and reduced physical fitness. Both study products elicited a moderately pleasant user experience, and the between-group difference was not significant. A clinically relevant improvement in exercise tolerance was observed after switching to the combustion-free products under investigation. Risk perception for conventional cigarettes was consistently higher than that for the combustion-free study products under investigation.

**Conclusions:**

Switching to HTPs elicited a marked reduction in cigarette consumption among people who smoke and do not intend to quit, which was comparable to refillable ECs. User experience and risk perception were similar between the HTPs and ECs under investigation. HTPs may be a useful addition to the arsenal of reduced-risk alternatives for tobacco cigarettes and may contribute to smoking cessation. However, longer follow-up studies are required to confirm significant and prolonged abstinence from smoking and to determine whether our results can be generalized outside smoking cessation services offering high levels of support.

**Trial Registration:**

ClinicalTrials.gov NCT03569748; https://clinicaltrials.gov/ct2/show/NCT03569748

## Introduction

According to the World Health Organization, smoking is endemic, with more than 1.1 billion smokers worldwide, resulting in approximately 7 million premature deaths every year [1]. Deaths are primarily due to lung cancer and fatal complications of ischemic heart disease and chronic obstructive pulmonary disease (COPD) [1-3]. The risk of developing diseases has been shown to significantly reduce when stopping smoking [4,5].

Achieving cessation is challenging, because quit rates are low, relapse rates are high, and many smokers wish to continue to smoke [6,7]. The compulsion to smoke is difficult to break, and even for those who do quit smoking, relapse is the norm. For unsupported quit attempts, 80% of people relapse in the first month [7,8], and among people who smoke and use treatments, 75% fail within 6 months, with the large majority resuming smoking within 2 weeks [9]. Even among those who quit smoking during hospitalization and intended to stay quit, 25% relapsed on the first day after discharge [10].

Substitution of combustible tobacco cigarettes with less harmful combustion-free nicotine delivery alternatives (NDAs), such as electronic cigarettes (ECs) and heated tobacco products (HTPs), is now a relatively new option available to smokers [6,11-13].

ECs operate by heating an element that vaporizes a solution. HTPs consist of a holder that electronically transfers controlled heat to tobacco sticks that generate a nicotine-containing aerosol. Since the emission aerosols of combustion-free NDAs are produced at a much lower vaporizing/heating temperature compared to that of combustion (which generally starts above 400 °C), they contain less harmful and potentially harmful chemicals than tobacco smoke [14-19].

Although not completely risk free, EC and HTP use may help respiratory patients to achieve sustained abstinence from cigarette smoking, with clinically relevant health gains [20,21]. The most recent Cochrane review concluded that ECs with nicotine increased quit rates, and compared to nicotine replacement therapy, a risk ratio (RR) of 1.53 was reported, indicating, in absolute terms, an additional 3 quitters for every 100 using ECs [22]. However, formal demonstration of the efficacy of HTPs for smoking cessation is not yet available [23].

HTPs often mimic a hand-to-mouth experience that is very similar to that of conventional tobacco cigarettes. By mimicking the experience of tobacco smoking and its associated rituals, the use of HTPs can provide adequate compensatory physical and behavioral effects [24,25], likely serving as an effective method of relapse prevention [21].

It is not clear if HTPs provide a more gratifying smoking experience compared to ECs, and a direct comparison between the 2 types of products has never been investigated. With this in mind, we conducted a prospective randomized noninferiority switching trial to compare effectiveness, tolerability, and product satisfaction/adoption between HTPs and refillable ECs among people who smoke and do not intend to quit.

## Methods

### Study Participants

Eligibility criteria have been described previously in detail [26]. In brief, adult people smoking ≥10 cigarettes per day for the past year, having an exhaled carbon monoxide (eCO) level of ≥7 ppm, not intending to quit in the next 30 days, and interested in switching to combustion-free NDAs were recruited among hospital/university staff, via social media, or through word of mouth. Unwillingness to quit was confirmed by the answer “No” to the following questions: “Do you plan to quit smoking within the next 30 days?” and “Do you wish to participate in a smoking cessation program?” The exclusion criteria were as follows: (1) history of depression, panic disorder, psychosis, or bipolar disorder; (2) significant history of alcoholism or drug/chemical abuse within 12 months prior to screening; (3) known clinically significant diseases that, in the opinion of the investigator, would jeopardize the safety of the participant or impact the validity of the study results; (4) use of any tobacco/nicotine delivery device (except for own brands of cigarettes) within the last 3 months; and (5) use of nicotine replacement therapy or other smoking cessation therapies within the last 3 months.

### Trial Design and Study Visits

Details of the study design and protocol have been previously published [26]. In brief, this was a 12-week, randomized, 2 parallel arm, open-label, noninferiority trial conducted to compare effectiveness, tolerability, adoption rates, and acceptability between HTPs and ECs in regular smokers (Multimedia Appendix 1). The trial consisted of a total of 8 study visits (see Multimedia Appendix 1). Participants could choose 1 out of 3 different flavors for each class of products and were provided with their preferred flavor for the whole duration of the study. Motivational counseling [27,28] was offered throughout the study to maximize study product adherence, to favor transition away from combustible tobacco cigarettes, and to prevent relapse back to smoking. Activities carried out during study visits are detailed in Multimedia Appendix 2.

**Figure 1:**
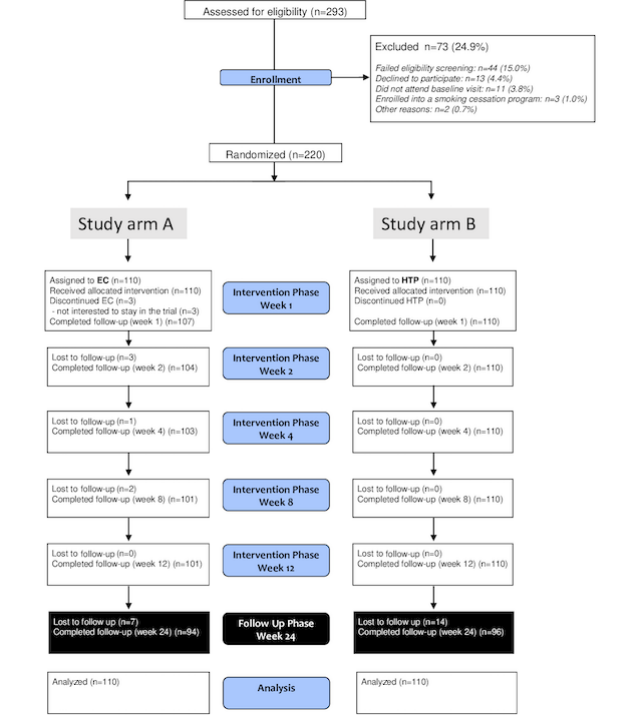
CONSORT (Consolidated Standards of Reporting Trials) flow diagram. EC: electronic cigarette; HTP: heated tobacco product.

The study was conducted in accordance with the Guideline for Good Clinical Practice and followed the Consolidated Standards of Reporting Trials (CONSORT) reporting guidelines for randomized studies (Figure 1).

### Study Products

HTPs and ECs were provided for the whole duration of the intervention phase (12 weeks).

#### HTPs

Participants randomized to the HTP arm of the study received IQOS 2.4 Plus consisting of a pen-like holder into which a tobacco stick is inserted and heated, and a battery case to recharge the holder after each use. IQOS 2.4 Plus was the only HTP available on the Italian market when this trial was designed. The device is to be used with tobacco sticks specifically processed and manufactured for IQOS (named *HEETS*). Participants could choose from 3 varieties of tobacco sticks (*HEETS* Amber, rich tobacco; *HEETS* Yellow, smooth tobacco; and *HEETS* Turquoise, menthol-flavored tobacco), which were available for sale on the Italian market at the time of the study.

#### ECs

Participants randomized to the EC arm of the study received JustFog Q16 Starter Kit consisting of a battery and a 1.9-mL refillable tank fitted with a 1.6-Ohm nichrome coil. Participants could choose from 3 varieties of e-liquid flavors (Puff Riserva Country 16 mg, sweet tobacco flavor; Puff Riserva Toscana 16 mg, full tobacco flavor; and Puff Artic 16 mg, menthol flavor; all 3 are formulated in 50% propylene glycol/40% vegetable glycerin/10% H_2_O), which were chosen for the trial by an expert panel to match the sensory experiences of the 3 IQOS tobacco sticks selected for the study.

### Study Endpoints

The primary efficacy endpoint of the study was the continuous abstinence rate (CAR) from week 4 to week 12 (CAR weeks 4-12). Abstinence from smoking was defined as eCO-verified (<10 ppm) self-reported abstinence from cigarette smoking. CAR weeks 4-12 was used to compare quit rates between IQOS-HTP and JustFog-EC.

The secondary efficacy endpoints were the 7-day point prevalence of abstinence at week 12 and the continuous reduction rate (CRR) from week 4 to week 12 (CRR weeks 4-12). Smoking reduction was self-reported. A reduction in the number of cigarettes smoked per day of 50% from baseline was considered of importance and excluded those labeled as CAR weeks 4-12. The CRR weeks 4-12 was used to compare reduction rates between IQOS-HTP and JustFog-EC. Participants who could not be classified as CAR weeks 4-12 or CRR weeks 4-12 were considered to have failed. Safety reporting details have been previously published [26].

Product satisfaction was investigated by using the following questionnaires adapted and validated for EC and HTP use: modified Cigarette Evaluation Questionnaire (mCEQ) and modified Smoking Cue Appeal Survey (mSCAS). Risk perception was assessed by using the Perceived Risk Instrument for conventional cigarettes (PRI-P CC) and the Perceived Risk Instrument for reduced risk products (PRI-P RRP). The effect on quality of life was investigated by questionnaires (ie, EQ-5D-5L and EQ VAS) and by measuring changes in body weight and exercise tolerance (ie, V̇O_2_ max by the Chester step test). Self-reported EC or HTP use at each study visit was verified against the product use check and reported in the electronic case report form (e-CRF). The product use check was used to calculate daily consumption.

### Study Assessments

The assessments carried out during study visits are listed in Multimedia Appendix 2 and included the following: (1) number of cigarettes smoked per day; (2) adverse events; (3) eCO levels evaluated with a calibrated handheld device (MicroCO); (4) resting blood pressure and heart rate taken with a semiautomated oscillometric sphygmomanometer (Smart Pressure, CA-MI); (5) body weight, height, body fat, visceral fat, fat-free mass, body muscle mass, bone body mass, metabolic age, and water content taken with a body composition analyzer (Tanita SC-240, Tanita); (6) BMI calculated by dividing weight by height square (kg/m^2^); and (7) Chester step test to determine maximal aerobic capacity (ie, V̇O_2_ max).

Other measurements included the following questionnaires: (1) Fagerstrom Test for Cigarette Dependence [29]; (2) mCEQ [30]; (3) mSCAS [31]; (4) PRI-P CC and PRI-P RRP [32]; and (5) EQ-5D-5L and EQ VAS, a standardized measure of health-related quality of life [33].

Secondary analyses of blood pressure, heart rate, BMI, and Chester step test results by smoking phenotype classification will be reported in separate papers.

### Ethical Considerations

The Ethical Review Board of Azienda Ospedaliero Universitaria “Policlinico-V. Emanuele,” Università di Catania, Italy, reviewed and approved the study (approval reference number: 215/2017/PO). All participants provided written informed consent prior to participation in the study. The study has been registered at ClinicalTrial.gov (trial registration ID: NCT03569748). Study data are deidentified, and participants did not receive compensation.

### Statistical Methods

A detailed description of the sample size calculation can be found in the published research protocol [26].

All the analyses were performed using SAS Version 9.4 (SAS Institute Inc). The primary efficacy endpoint of the study in the experimental study group was calculated with a noninferiority threshold of 15%. An α level of .05 was considered. Quit rates were evaluated on an intention-to-treat basis. CAR weeks 4-12 percentages and odds ratios (ORs) were calculated and used to compare quit rates between the IQOS-HTP and JustFog-EC study groups with the chi-square test. Moreover, CRR weeks 4-12 percentages and ORs were calculated and used to compare reduction rates between the IQOS-HTP and JustFog-EC study groups with the chi-square test. For the 7-day point prevalence of smoking abstinence and reduction, percentages were calculated at each study visit to illustrate trends.

Safety data were presented as descriptive statistics separately by study group. Any events documented in the period from the point of product randomization (V1) until the end of the intervention phase at 12 weeks when study products were withdrawn (V6) were considered as relevant for safety analysis.

Descriptive statistics of product acceptability measures (ie, mCEQ and mSCAS) were presented as summary tables by study group and study visit. Changes in mCEQ and mSCAS scores within and between study groups were analyzed using the Wilcoxon signed rank test and Wilcoxon rank sum test, respectively.

For risk perception, descriptive statistics of PRI-P CC and PRI-P RRP values were presented as summary tables by study group and study visit. Changes in PRI-P scores within and between study groups were analyzed using the Wilcoxon signed rank test and Wilcoxon rank sum test, respectively.

EQ-5D-5L, EQ VAS, body weight, and exercise tolerance were presented as descriptive statistics, and within- and between-group comparisons were carried out using the Wilcoxon signed rank test and Wilcoxon rank sum test, respectively.

A multiple logistic regression model was prepared to identify variables able to influence the primary outcome CAR weeks 4-12. We performed an a priori selection of variables able to act as determinants, effect modifiers, or confounders of quitting success. The continuous variables were categorized according to cutoffs based clinically. The univariate analysis was performed considering the subgroups CAR and no CAR as the outcome. The following factors, for which a statistical difference was detected, were included in the model: gender, daily cigarette consumption, and 4 psychological aspects included as domains in the questionnaires (ie, product satisfaction, psychological reward, enjoyment, and craving).

## Results

### Baseline Characteristics

The CONSORT flow diagram of the study subjects is shown in Figure 1. A total of 220 smokers were enrolled in the study, with 211 (95.9%) participants completing the intervention phase. Baseline characteristics of the participants are shown by study product assignment in Table 1 and were comparable between the study groups. On average, participants were Caucasian adults (approximately 41 years old), were mostly men (approximately 57.3%), had smoked about a pack daily for approximately 24 years, had a moderate Fagerstrom Test for Cigarette Dependence score of about 5, and had an average of 2 quit attempts in the past.

**Table 1:** Baseline characteristics of the participants.

Characteristic			HTP^a^ group (N=110)		EC^b^ group (N=110)		*P* value
**Sex, n**							.06
	Female	56 (50.9)		70 (63.6)			
	Male	54 (49.1)		40 (36.4)			
Age (years), mean (SD)			41.3 (16.1)		41.3 (16.9)		.97
**Education level, n (%)**							.84
	Primary school	2 (1.8)		1 (0.9)			
	Secondary school	20 (18.2)		24 (21.8)			
	High school	64 (58.2)		61 (55.5)			
	University	24 (21.8)		24 (21.8)			
Cigarettes per day, mean (SD)			22.6 (10.1)		22.8 (10.9)		.91
Exhaled carbon monoxide (ppm), mean (SD)			26.2 (13.6)		26.9 (15.4)		.71
Fagerstrom Test for Cigarette Dependence score			5.8 (2.1)		6.0 (2.2)		.47
Years of smoking, mean (SD)			24.5 (15.6)		22.9 (16.0)		.44
Number of quit attempts, mean (SD)			1.9 (2.7)		1.9 (2.5)		.96

^a^HTP: heated tobacco product.

^b^EC: electronic cigarette.

### Smoking Abstinence and Reduction Rates

**Figure 2:**
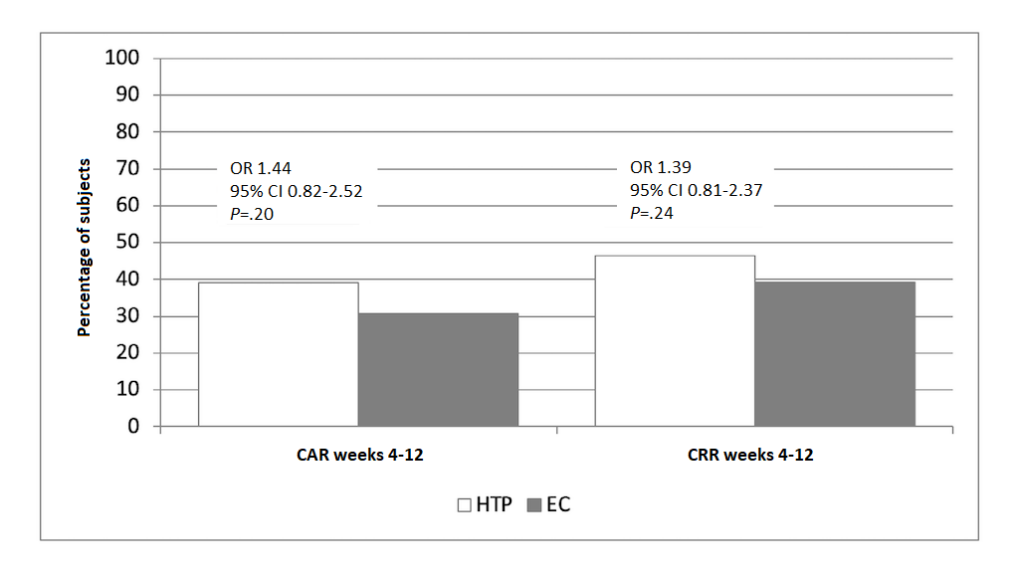
Smoking abstinence rates and smoking reduction rates. CAR: continuous abstinence rate; CRR: continuous reduction rate; EC: electronic cigarette; HTP: heated tobacco product.

**Figure 3:**
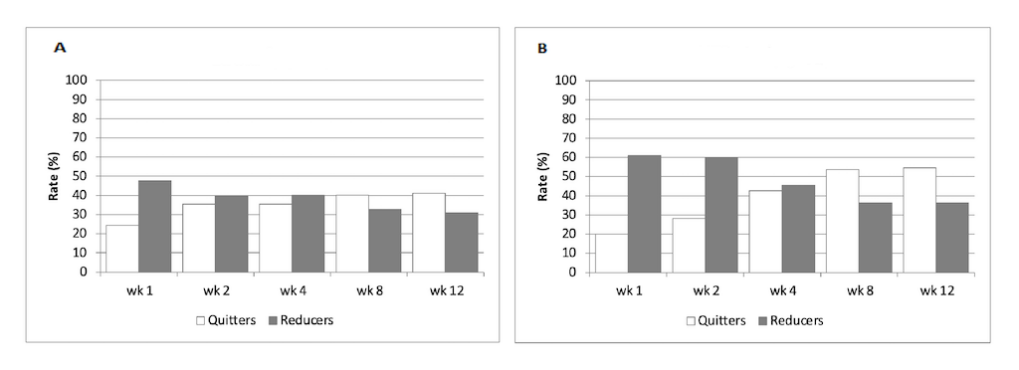
Seven-day prevalence of smoking abstinence and reduction in the electronic cigarette (A) and heated tobacco product (B) study groups.

Smoking abstinence rates (CAR weeks 4-12), reduction rates (CRR weeks 4-12), and 7-day point prevalence of smoking abstinence and reduction are shown in Figure 2, and Figures 3A and 3B.

High quit rates, evaluated on an intention-to-treat basis, were observed for both study groups; the CAR weeks 4-12 values for IQOS-HTP and JustFog-EC were 39.1% (43/110) and 30.8% (33/107), respectively (Figure 2). The between-group difference for the CAR weeks 4-12 was not significant (*P*=.20; chi-square test) and satisfied the noninferiority criteria of the study that differences in quit rates between products did not exceed 15% (OR 1.44, 95% CI 0.82-2.52).

High reduction rates, assessed among participants who were still smoking, were also reported, and the CRR weeks 4-12 values for IQOS-HTP and JustFog-EC were 46.4% (51/110) and 39.3% (42/107), respectively (Figure 2). The between-group difference for the CRR weeks 4-12 was not significant (*P*=.24; chi-square test; OR 1.39, 95% CI 0.81-2.37).

The 7-day point prevalence of smoking abstinence was >20% throughout the intervention phase, with values peaking at week 8 (V5) and week 12 (V6) (Figures 3A and 3B). The 7-day point prevalence of smoking abstinence values for IQOS-HTP and JustFog-EC were 53.6% (59/110) and 40.2% (43/107) at week 8, and 54.5% (60/110) and 41.1% (44/107) at week 12, respectively.

The 7-day point prevalence of smoking reduction (ie, dual use) was higher during the first 2 weeks of the intervention phase (Figures 3A and 3B). The 7-day point prevalence of smoking reduction values for IQOS-HTP and JustFog-EC were 60.9% (67/110) and 47.7% (51/107) at week 1, and 60.0% (66/110) and 39.3% (42/107) at week 2, respectively.

For both study products, Figures 3A and 3B also show progressive reduction in the proportion of dual use in the study, which was paralleled by rising prevalence of exclusive single use by the end of the intervention phase.

### Product Preference, Acceptability, and Risk Perception

Among participants in the EC study arm, 50.9% (56/110) chose Puff Riserva Country, 30.9% (34/110) chose Puff Riserva Tuscan, and 18.2% (20/110) chose Puff Artic e-liquid. Among participants in the HTP study arm, 56.4% (62/110) chose *HEETS* Amber, 33.6% (37/110) chose *HEETS* Yellow, and 10.0% (11/110) chose *HEETS* Turquoise tobacco sticks. Technical issues (eg, device malfunctions) were relatively uncommon (Multimedia Appendix 3).

Appeal of the study products was analyzed using the mCEQ and mSCAS. No significant within-group changes in the mCEQ and mSCAS scores were observed (Wilcoxon signed rank test; Multimedia Appendix 4). Between-group changes were also not significantly different (Wilcoxon rank sum test; Multimedia Appendix 4). Moderate liking of the study products, mild psychological reward, moderate enjoyment of the respiratory tract sensation, and craving reduction with minimal aversion were noted (Multimedia Appendix 4). The mSCAS showed that the study products elicited a moderately pleasant user experience (Multimedia Appendix 4).

As expected, risk perception for conventional cigarettes was consistently higher than for the combustion-free study products (Multimedia Appendices 4 and 5). Within-group changes in PRI-P CC scores were small but significantly higher for both study groups (IQOS-HTP, *P*<.001; JustFog-EC, *P*=.003; Wilcoxon signed rank test). Between-group comparisons were not statistically significant (Wilcoxon rank sum test). No significant within- or between-group changes were observed for PRI-P RRP.

Consumption patterns of conventional tobacco cigarettes, vaping products, and HTPs throughout the study are illustrated in Multimedia Appendices 6 and 7.

### Evaluation of Participant Well-being

EQ-5D-5L and EQ VAS results are summarized in Multimedia Appendix 8. No significant changes were observed between the 2 study groups. Within-group analyses for both IQOS-HTP and JustFog-EC showed small but significant changes in all EQ-5D-5L domains, with the exception of domain 2. Regarding EQ VAS, within-group analyses for both IQOS-HTP and JustFog-EC showed small but significant changes from baseline (IQOS-HTP and JustFog-EC; *P*<.001 for all comparisons).

**Figure 4:**
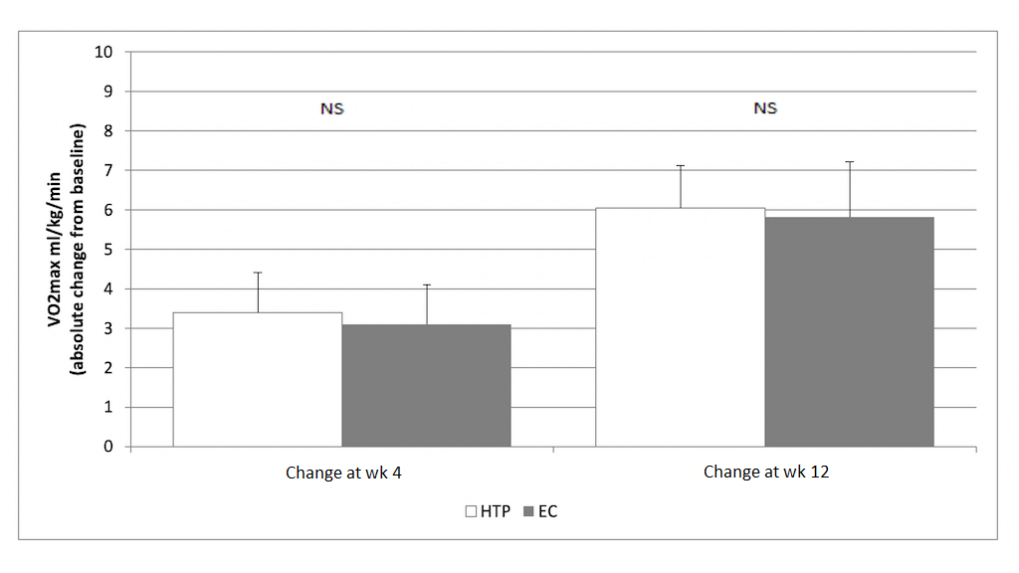
Chester step test results. EC: electronic cigarette; HTP: heated tobacco product; NS: not significant.

Changes in exercise tolerance between study products were not significant (Figure 4; Multimedia Appendix 8). However, a significant improvement from baseline was observed after switching to the combustion-free products under investigation. For JustFog-EC, reported changes of 2.6 and 7.0 mL/kg/min were noted at week 4 and week 12, respectively (*P*<.001), while for IQOS-HTP, reported changes of 3.4 and 6.4 mL/kg/min were noted at week 4 and week 12, respectively (*P*=.007) (Figure 4; Multimedia Appendix 8). These V̇O_2_ max improvements were consistently greater than the minimum clinically important difference (MCID) defined as an improvement in the anaerobic threshold of at least 2 mL O_2_/kg/min.

### Predictors of Smoking Abstinence

The results of the abovementioned logistic regression model showed the following evidence: males were less likely to achieve CAR weeks 4-12 compared to females (OR 0.457, 95% CI 0.249-0.840), subjects who had a high consumption of the product were likely to achieve CAR weeks 4-12 compared to those who had a low consumption of the product (OR 0.450, 95% CI 0.212-0.956), and subjects who had a high craving reduction were likely to achieve CAR weeks 4-12 compared to those who had a low craving reduction (OR 0.391, 95% CI 0.186-0.825).

### Adverse Events

The reported numbers of adverse events are listed in Multimedia Appendix 9. Most adverse events were rated as mild or moderate and did not led to discontinuation of product use in either study group. In general, the most commonly reported adverse events were cough and reduced physical fitness. Oropharyngeal irritation and dyspnea were more frequent in the EC group than in the HTP group. No serious adverse events were reported during the study. No significant changes in the mean resting heart rate, blood pressure, and BMI during product use were observed between and within study groups.

## Discussion

HTP use elicited a marked reduction in cigarette consumption, resulting in almost 40% abstinence from smoking by the end of the study. When present, adverse events were mild and transitory. This is the first study to directly compare ECs to HTPs, showing comparable effectiveness and tolerability between the JustFog-EC and IQOS-HTP study groups.

RCT findings for IQOS-HTP are in agreement with the findings of an American Cancer Society analysis that found a substantial decline in cigarette sales after the introduction of IQOS in Japan [34] and an observational study of COPD patients that reported substantially attenuated or ceased cigarette consumption in the long term after switching to IQOS [21], but are different from the findings of a recent online survey of Korean adults showing a low probability of quitting among IQOS users [35].

Multiple factors contributed to the high quit rate observed among IQOS-HTP and JustFog-EC users: (1) participants were keen to switch to combustion-free NDAs; (2) personalized motivational counseling was administered by psychologists proficient in both smoking cessation and harm reduction at each study visit; (3) top selling products in their respective category (ie, JustFog for ECs and IQOS for HTPs) were given for free in the study and most participants found these products appealing; (4) study products had a pharmacokinetic profile of nicotine uptake mimicking that of conventional cigarettes [36,37]; (5) participants perceived the study products as less harmful than their own cigarettes; and (6) regular use of study products relieved cigarette-induced symptoms and improved exercise tolerance.

The same factors might have contributed to the comparable effectiveness between ECs and HTPs and to the progressive transition from dual use to solo use by the end of the intervention phase. Dual use is known for being a common transitory state, with transitions to solo use taking variable time to occur [38,39]. In our switching trial, IQOS users reported increased dual use in the first 2 weeks compared to JustFog users, but this quickly stabilized by week 4. This could indicate different learning curves for the 2 products.

For this study, we selected the top selling products in their respective category (ie, ECs and HTPs). Both IQOS-HTP and JustFog-EC performed well in the study, as technical issues (eg, malfunctions) were relatively uncommon. Participants enjoyed using both study products, with mCEQ scores showing mild to moderate positive responses in terms of product acceptability, cigarette craving reduction, and physical and psychological reward; mSCAS scores indicating a moderately pleasant user experience; and consumption data revealing regular constant product use throughout the study. This is consistent with the notion that a positive sensorial experience and product enjoyment can contribute to the effectiveness of combustion-free products in terms of cessation outcomes [40-42]. Analyses of PRI-P scoring showed that the study products were perceived to be much less harmful than combustible cigarettes, confirming findings from previous studies [43,44]. IQOS-HTP was perceived to be slightly riskier than JustFog-EC, and in the authors’ opinion, this is probably because IQOS shows marked similarities with conventional cigarettes. Moreover, regular use of the study products provided adequate control of cravings (thereby serving as an effective method of relapse prevention), reduced symptoms, and had an overall positive impact on physical fitness, with similar improvements for IQOS-HTP and JustFog-EC. This may also explain why the trend in quit rates increased over time in this switching study; this is discordant to what is generally observed in standard smoking cessation studies in which success rates decline over time.

Adverse events were mild and did not led to discontinuation of product use in either study group. None of the participants abused the products under investigation in terms of excessive daily consumption. In some participants, HTP use was associated with mild cough and reduced physical fitness in line with previous observations [23]. However, the frequency of these symptoms was much lower by the end of the study compared to baseline. Previous smoking history is a key confounder when evaluating the health effects of combustion-free nicotine alternatives in switching studies, as shown by the progressive reduction in the frequency of symptoms by the end of this study. Oropharyngeal irritation was more frequent in the EC group than in the HTP group, probably because of the relatively high level of propylene glycol (a respiratory irritant) in the vaping products under investigation (formulated in 50% propylene glycol/40% vegetable glycerin/10% H_2_O). This common irritative response has been shown to be transient and is of uncertain prognostic value [45].

A clinically relevant improvement in exercise tolerance was observed after switching to the combustion-free products under investigation as early as 4 weeks. Greater improvement was observed at 12 weeks as there was a much higher prevalence of quitters by the end of the intervention phase compared to 4 weeks. This is in agreement with the improvement in the level of exercise tolerance shown in prospective studies of COPD patients who switched to ECs [20] and HTPs [21]. The time-dependent improvement in exercise tolerance that occurs after switching may be explained by the marked decline in carbon monoxide exposure and in carboxyhemoglobin levels following cigarette substitution with combustion-free alternatives [46,47].

The trial had strengths and limitations. First, among the innovative features of this randomized controlled switching study, adherence to the study products was enhanced by offering a selection of different products to choose from according to preference/liking. Three aromas of tobacco sticks and three e-liquid flavors were provided to best match participants’ sensorial experiences. Nonetheless, these choices remain limited (ie, only 3 different flavors for each class of products) and product specific, thus reducing the generalizability of the study findings. In addition, multiple flavor use is common among e-cigarette users, and switching between flavors is frequently reported even during daily use [48,49]. Du et al also reported that only 1.8% of regular e-cigarette users were using only 1 flavor on a regular basis [50]. Thus, the effectiveness of vaping products for smoking substitution may be further improved.

Second, after close scrutiny, we chose to offer the best vaping devices and HTPs available on the Italian market at the time of the study. More details about the selection process have been published previously [26]. Obviously, product assignment could not be blinded, and strong product preference (IQOS-HTP vs JustFog-EC) could have introduced an allocation bias. Only 3 subjects dropped out soon after randomization when they learned that their product allocation (ie, JustFog-EC) was not their preferred one (ie, IQOS-HTP). However, we cannot exclude that if JustFog-EC was seen as an inferior option, participants in this study group might have put less effort into their switching attempt than those allocated to IQOS-HTP. Nonetheless, the CARs in the JustFog-EC group were at least as high as previously reported [22].

Third, study products were provided in combination with personalized motivational counseling administered by psychologists proficient in both smoking cessation and harm reduction. Provision of expert guidance in the context of a switching trial conducted at specialized smoking cessation services may limit the generalizability of the study findings. Changes in tobacco/nicotine use behavior and product use will be investigated in a separate follow-up study under real-life conditions.

In conclusion, this study confirmed the effectiveness of ECs for cigarette substitution and smoking cessation [22,51], and revealed for the first time that HTP use can promote abstinence from cigarette smoking in combination with motivational counseling. HTPs provided a comparable experience to ECs. Moreover, these results were paralleled by a marked reduction in reported symptoms. Based on the findings of this study, HTPs may represent a valuable addition to the arsenal of reduced-risk products in terms of their smoking substitution potential, but longer follow-up studies are required to confirm significant and prolonged abstinence from smoking and to determine whether our results can be generalized outside smoking cessation services offering high levels of support.

## Appendix

Multimedia Appendix 1 Study diagram.

Multimedia Appendix 2 Study schedule and assessments.

Multimedia Appendix 3 Detailed technical issues.

Multimedia Appendix 4 Summary of the measures of product preference, acceptability, and risk perception.

Multimedia Appendix 5 Risk perception.

Multimedia Appendix 6 Consumption data for study participants (per protocol population).

Multimedia Appendix 7 Average daily consumption.

Multimedia Appendix 8 Summary of the measures of participant well-being.

Multimedia Appendix 9 Adverse Events.

Multimedia Appendix 10 CONSORT 2010 checklist.

## Abbreviations

CAR: continuous abstinence rate
CONSORT: Consolidated Standards of Reporting Trials
COPD: chronic obstructive pulmonary disease
CRR: continuous reduction rate
EC: electronic cigarette
eCO: exhaled carbon monoxide
HTP: heated tobacco product
mCEQ: modified Cigarette Evaluation Questionnaire
mSCAS: modified Smoking Cue Appeal Survey
NDA: nicotine delivery alternative
OR: odds ratio
PRI-P CC: Perceived Risk Instrument for conventional cigarettes
PRI-P RRP: Perceived Risk Instrument for reduced risk products

## Data Availability

We may share deidentified individual participant-level data that underlie the results reported in this article. Data will be available on receipt of a request detailing the study hypothesis and statistical analysis plan. All requests should be sent to the corresponding author. Based on the scientific rigor of the proposal, the study authors will discuss all requests and decide whether data sharing is appropriate. All applicants will be asked to sign a data access agreement.
