# Supplementary figures and images for: Comparing the Effectiveness, Tolerability, and Acceptability of Heated Tobacco Products and Refillable Electronic Cigarettes for Cigarette Substitution (CEASEFIRE): Randomized Controlled Trial
Source: JMIR Public Health Surveill. 2023 Apr 4;9:e42628. doi: 10.2196/42628 (PMC10131829; doi:10.2196/42628)

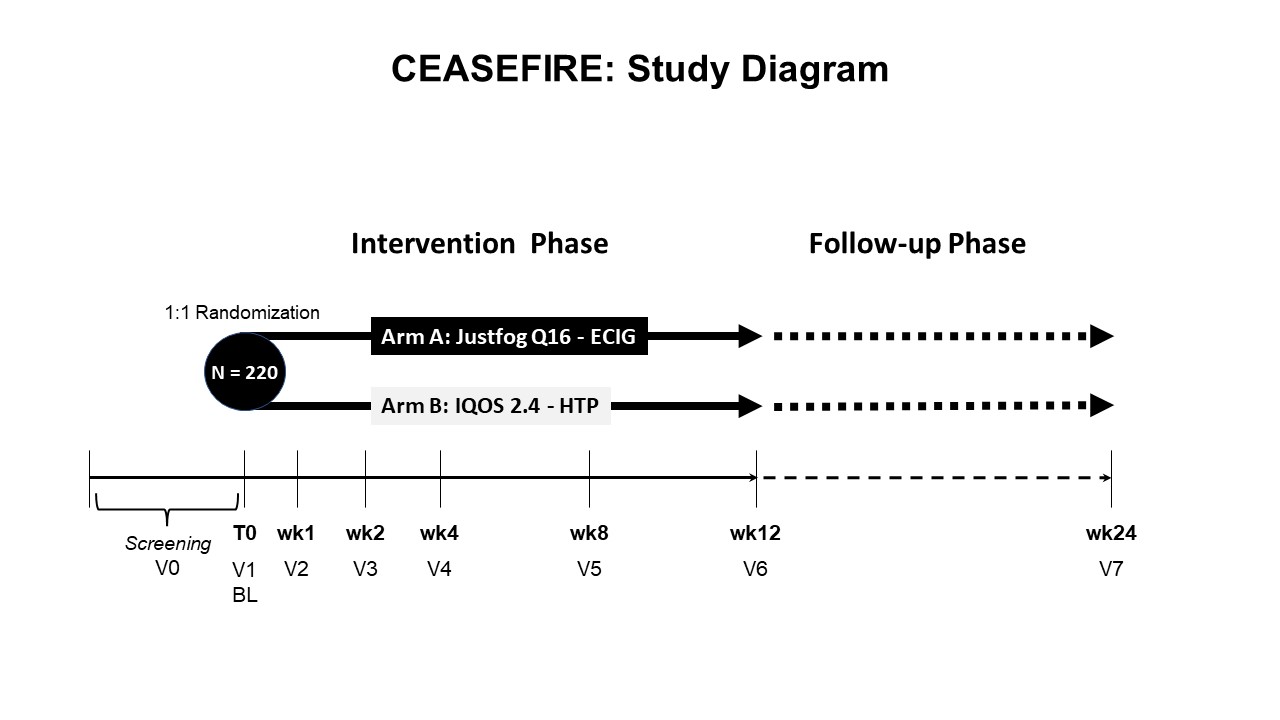

Supplement: Multimedia Appendix 1 [file publichealth_v9i1e42628_app1.docx]

**Multimedia Appendix 5.** Risk perception.


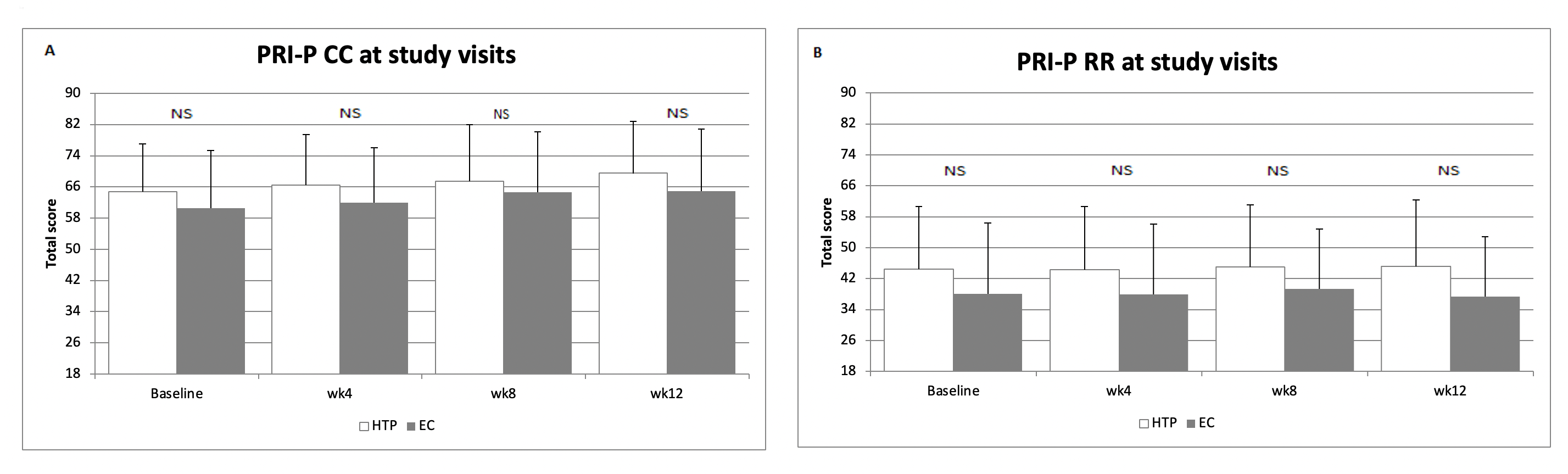

Supplement: Multimedia Appendix 5 [file publichealth_v9i1e42628_app5.docx]

**Multimedia Appendix 7.** Average daily consumption.

**
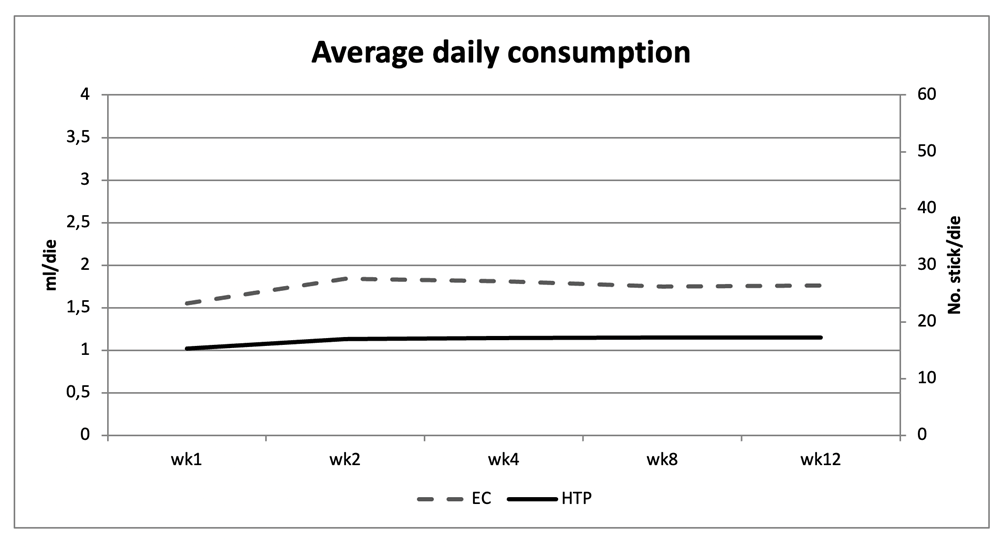
**

Supplement: Multimedia Appendix 7 [file publichealth_v9i1e42628_app7.docx]
