# Supplementary material for: Comparing the Effectiveness, Tolerability, and Acceptability of Heated Tobacco Products and Refillable Electronic Cigarettes for Cigarette Substitution (CEASEFIRE): Randomized Controlled Trial
Source: JMIR Public Health Surveill. 2023 Apr 4;9:e42628. doi: 10.2196/42628 (PMC10131829; doi:10.2196/42628)
Supplement: Multimedia Appendix 2 [file publichealth_v9i1e42628_app2.docx]

**Multimedia Appendix 2.** Study schedule and assessments.

| Procedure | Screen | BL | Week  1 | Week  2 | Week  4 | Week  8 | Week  12 | Week  24 |
| --- | --- | --- | --- | --- | --- | --- | --- | --- |
|  | V0 | V1 | V2 | V3 | V4 | V5 | V6 | V7 |
| Informed consent |  | X |  |  |  |  |  |  |
| Eligibility criteria | X | X |  |  |  |  |  |  |
| Randomization |  | X |  |  |  |  |  |  |
| Products familiarization |  | X |  |  |  |  |  |  |
| Smoking/vaping* history | X | X |  |  |  |  |  |  |
| Cigarette consumption | X | X | X | X | X | X | X | X |
| Exhaled CO |  | X | X | X | X | X | X | X |
| FTCD |  | X |  |  |  |  |  |  |
| BP, HR |  | X | X | X | X | X | X | X |
| Weight, height (BMI) |  | X |  |  |  |  | X | X |
| Instructions for urine collection and storage | X |  |  | X |  |  |  |  |
| Urine sample for processing |  | X |  |  | X |  |  |  |
| Tailored Motivational Interviewing counselling |  | X | X | X | X | X | X** |  |
| Adverse events |  | X | X | X | X | X | X |  |
| Chester Step Test |  | X |  |  | X |  | X | X |
| mCEQ |  |  |  |  | X | X | X | X |
| mSCAS |  |  |  |  | X | X | X | X |
| Perceived risk questionnaires |  | X |  |  | X | X | X | X |
| EQ-5D-5L & EQ-VAS |  | X |  |  | X | X | X | X |
| 1 week product supply (tobacco sticks for iQOS; e-liquid refill bottles for JustFog) |  | X | X |  |  |  |  |  |
| 2 weeks product supply (tobacco sticks for iQOS; e-liquid refill bottles for JustFog) |  |  |  | X |  |  |  |  |
| 4 weeks product supply (tobacco sticks for iQOS; e-liquid refill bottles for JustFog) |  |  |  |  | X | X |  |  |
| Product use check |  |  | X | X | X | X | X | X*** |

*Screen: screening visit; BL: baseline; CO: carbon monoxide; BP: blood pressure; HR: heart rate; BMI: body mass index; FCTD: Fagerstrom Test for Cigarette Dependence; mCEQ: Modified Cigarette Evaluation Questionnaire; mSCAS: modified Smoking Cue Appeal Survey; EQ-5D & EQ-VAS, Euro Quality of life questionnaires & visual analog scale.*

**Includes previous use of heated tobacco products*

***participants are informed that they are free to use any tobacco/nicotine product they wish or cease their use between V6 and V7.*

****collection of product use between V6 and V7.*
