# Supplementary material for: Comparing the Effectiveness, Tolerability, and Acceptability of Heated Tobacco Products and Refillable Electronic Cigarettes for Cigarette Substitution (CEASEFIRE): Randomized Controlled Trial
Source: JMIR Public Health Surveill. 2023 Apr 4;9:e42628. doi: 10.2196/42628 (PMC10131829; doi:10.2196/42628)
Supplement: Multimedia Appendix 3 [file publichealth_v9i1e42628_app3.docx]

**Multimedia Appendix 3.** Detailed technical issues.

Technical issues were relatively uncommon ((e.g. malfunction): throughout the study, substitution of study device was offered on 16 occasions for ECs (10 faulty batteries and 6 leaking tanks) and 9 for HTPs (5 broken blades and 4 faulty batteries).
