# Supplementary material for: Comparing the Effectiveness, Tolerability, and Acceptability of Heated Tobacco Products and Refillable Electronic Cigarettes for Cigarette Substitution (CEASEFIRE): Randomized Controlled Trial
Source: JMIR Public Health Surveill. 2023 Apr 4;9:e42628. doi: 10.2196/42628 (PMC10131829; doi:10.2196/42628)
Supplement: Multimedia Appendix 4 [file publichealth_v9i1e42628_app4.docx]

**Multimedia Appendix 4.** Summary of the measures of product preference, acceptability, and risk perception.

| **Questionnaires** | ***Study Group A (switch to EC)*** | | | | | ***Study Group B (switch to HTP)*** | | | | |
| --- | --- | --- | --- | --- | --- | --- | --- | --- | --- | --- |
|  | *Baseline*  *Median (IQR)* |  | Wk4  *Median (IQR)* | Wk8  *Median (IQR)* | Wk12  *Median (IQR)* | *Baseline*  *Median (IQR)* |  | Wk4  *Median (IQR)* | Wk8  *Median (IQR)* | Wk12  *Median (IQR)* |
| mCEQ*  -Product Satisfaction  -Psychological Reward  -Aversion 5  -Respiratory Enjoyment3  -Craving Reduction4 |  |  | 12.0 (10;14)  16.0 (11;20)  4.0 (2;5)  4.0 (3;5)  4.0 (2;5) | 12.0 (9;15)  17.0 (12;21)  4.0 (2;6)  4.0 (2;5)  4.0 (2;6) | 12.0 (9;15)  16.0 (10;21)  4.0 (2;5)  4.0 (3;4)  4.0 (2;5) |  |  | 13.5 (10;15)  18.5 (13;21)  4.0 (2;6)  4.0 (3;5)  4.0 (3;5) | 13.0 (10;15)  18.0 (13;22)  4.0 (2;6)  4.0 (3;5)  4.0 (3;5) | 14.0 (11;15)  17.5 (11;21)  4.0 (2;6)  4.0 (3;5)  4.0 (3;5) |
| mSCAS** |  |  | -3.0 (-7;-1) | -3.0 (-7;-1) | -3.0 (-8;0) |  |  | -2.0 (-5;0) | -2.0 (-5;0) | -2.0 (-5;0) |
| PRI-P CC*** | 63.5 (55;70) |  | 64.0 (55;73) | 67.0 (57;76) | 68.0 (54;77) | 67.0 (57;74) |  | 67.5 (57;76) | 68.5 (59;78) | 72.0 (62;81) |
| PRI-P RRP*** | 37.0 (28;48) |  | 37.0 (28;47) | 37.0 (31;44) | 37.0 (28;46) | 45.0 (34;51) |  | 45.0 (34;51) | 42.5 (34;55) | 42.5 (34;54) |

**mCEQ: “Product Satisfaction” scores* *ranging from minimum 3 to maximum 21; “Psychological Reward” ranging from minimum 5 to maximum 35; “Aversion” ranging from minimum 2 to maximum 14; “Enjoyment of Respiratory Tract Sensations” and; “Craving Reduction” both ranging from minimum 1 to maximum 7.*

*** mSCAS: scores ranging from -24 (extremely pleasant) to + 24 (extremely unpleasant).*

**** PRI-P CC / PRI-P RRP: scores ranging from 18 (no risk) to 90 (very high risk).*
