# Supplementary material for: Comparing the Effectiveness, Tolerability, and Acceptability of Heated Tobacco Products and Refillable Electronic Cigarettes for Cigarette Substitution (CEASEFIRE): Randomized Controlled Trial
Source: JMIR Public Health Surveill. 2023 Apr 4;9:e42628. doi: 10.2196/42628 (PMC10131829; doi:10.2196/42628)
Supplement: Multimedia Appendix 6 [file publichealth_v9i1e42628_app6.docx]

**Multimedia Appendix 6.** Consumption data for study participants (per protocol population).

| Study Product | Study Group A (switch to EC) | | | | | | Study Group B (switch to HTP) | | | | | |
| --- | --- | --- | --- | --- | --- | --- | --- | --- | --- | --- | --- | --- |
|  | Base | Wk1 | Wk2 | Wk4 | Wk8 | Wk12 | Base | Wk1 | Wk2 | Wk4 | Wk8 | Wk12 |
| No. participants | 110 | 107 | 104 | 103 | 101 | 101 | 110 | 110 | 110 | 110 | 110 | 110 |
| **Cigarette consumption/day** | | | | | | | | | | | | |
| Mean  (SD) | 22.3  (10.7) | 7.6  (9.0) | 6.0  (7.9) | 5.7  (7.8) | 5.8  (7.8) | 6.1  (8.1) | 22.6  (10.3) | 7.1 (7.6) | 5.1  (6.6) | 4.7  (7.6) | 4.4  (7.3) | 4.0  (6.5) |
| Min | 10 | 0 | 0 | 0 | 0 | 0 | 10 | 0 | 0 | 0 | 0 | 0 |
| Max | 60 | 48 | 35 | 43 | 35 | 35 | 60 | 33 | 25 | 35 | 33 | 25 |
| **Tobacco Stick consumption/day** | | | | | | | | | | | | |
| Mean  (SD) | - | - | - | - | - | - | - | 15.3  (10.3) | 17.0  (10.0) | 17.1  (10.1) | 17.2  (10.2) | 17.2  (10.3) |
| Min | - | - | - | - | - | - | - | 0 | 0 | 0 | 0 | 0 |
| Max | - | - | - | - | - | - | - | 63 | 61 | 66 | 60 | 60 |
| **E-liquid consumption/day** | | | | | | | | | | | | |
| Mean  (SD) | - | 1.6  (1.1) | 1.8  (1.1) | 1.8  (1.3) | 1.7  (1.3) | 1.8  (1.4) | - | - | - | - | - | - |
| Min | - | 0 | 0 | 0 | 0 | 0 | - | - | - | - | - | - |
| Max | - | 4 | 5 | 6 | 6 | 6 | - | - | - | - | - | - |
