# Supplementary material for: Comparing the Effectiveness, Tolerability, and Acceptability of Heated Tobacco Products and Refillable Electronic Cigarettes for Cigarette Substitution (CEASEFIRE): Randomized Controlled Trial
Source: JMIR Public Health Surveill. 2023 Apr 4;9:e42628. doi: 10.2196/42628 (PMC10131829; doi:10.2196/42628)
Supplement: Multimedia Appendix 8 [file publichealth_v9i1e42628_app8.docx]

**Multimedia Appendix 8.** Summary of the measures of participant well-being.

| **Measures** | ***Study Group A (switch to EC)*** | | | | | ***Study Group B (switch to HTP)*** | | | | |
| --- | --- | --- | --- | --- | --- | --- | --- | --- | --- | --- |
|  | *Baseline*  *Median (IQR)* |  | Wk4  *Median (IQR)* | Wk8  *Median (IQR)* | Wk12  *Median (IQR)* | *Baseline*  *Median (IQR)* |  | Wk4  *Median (IQR)* | Wk8  *Median (IQR)* | Wk12  *Median (IQR)* |
| EQ-5D-5L*  -Mobility  -Self-Care  -Usual Activities  -Pain/Discomfort  -Anxiety/Depression | 1.3 (0.6)  1.1 (0.2)  1.3 (0.5)  1.6 (0.8)  1.9 (0.9) |  | 1.2 (0.5)  1.1 (0.3)  1.3 (0.5)  1.5 (0.7)  1.7 (0.8) | 1.2 (0.5)  1.0 (0.2)  1.2 (0.5)  1.4 (0.6)  1.6 (0.8) | 1.2 (0.5)  1.0 (0.2)  1.2 (0.4)  1.4 (0.7)  1.6 (0.9) | 1.3 (0.6)  1.0 (0.1)  1.2 (0.5)  1.6 (0.7)  1.8 (0.8) |  | 1.2 (0.6)  1.1 (0.4)  1.1 (0.4)  1.4 (0.8)  1.7 (0.8) | 1.2 (0.5)  1.0 (0.2)  1.1 (0.3)  1.3 (0.6)  1.6 (0.7) | - 1. (0.4)   2. (0.3)   3. (0.3)   1.4 (0.7)  1.6 (0.8) |
| EQ VAS** | *75.5 (18.1)* |  | 78.6 (15.5) | 81.7 (13.7) | 82.1 (15.0) | *75.6 (15.1)* |  | 79.2 (14.3) | 81.8 (13.4) | 82.6 (13.4) |
| VO2MAX (ml/kg/min)*** | 43.9 (3.0) |  | 46.5 (3.1) |  | 50.9 (3.2) | 38.3 (2.7) |  | 41.7 (2..9) |  | 44.7 (2.8) |

**EQ-5D-5L: levels of impairment with scores ranging from minimum 1 (“no problems”) to maximum5 (“extreme problems” or “unable to do”).*

***EQ VAS: scores ranging from 0 (“worst imaginable health”) to 100 (“best imaginable health”).*

****VO2MAX: according to normative data (age range 20-49 years old) values ranging from 42-50 and from 35-43 are considered “good” for males and females, respectively (reference: Heyward V.H. 2006). The minimum clinically important difference for VO2MAX is defined as an improvement in anaerobic threshold of > 2 ml O2/kg/min.*
