# Supplementary material for: Comparing the Effectiveness, Tolerability, and Acceptability of Heated Tobacco Products and Refillable Electronic Cigarettes for Cigarette Substitution (CEASEFIRE): Randomized Controlled Trial
Source: JMIR Public Health Surveill. 2023 Apr 4;9:e42628. doi: 10.2196/42628 (PMC10131829; doi:10.2196/42628)
Supplement: Multimedia Appendix 9 [file publichealth_v9i1e42628_app9.docx]

**Multimedia appendix 9: Most commonly reported Adverse Events**

| **Adverse Events** | **Study Group A (switch to EC)** | | | | | | **Study Group B (switch to HTP)** | | | | | |
| --- | --- | --- | --- | --- | --- | --- | --- | --- | --- | --- | --- | --- |
|  | BL | Wk1 | Wk2 | Wk4 | Wk8 | Wk12 | BL | Wk1 | Wk2 | Wk4 | Wk8 | Wk12 |
| Cough* | 22 (20%) | 9  (8.4%) | 6 (5.8%) | 3 (2.9%) | 3 (3%) | 3  (3%) | 25 (22.7%) | 10 (9.1%) | 9 (8.2%) | 3 (2.7%) | 3 (2.7%) | 2 (1.8%) |
| Reduced physical fitness | 19 (17.3%) | 6 (5.6%) | 4 (3.8%) | 1 (1%) | 0 | 1  (1%) | 27 (24.6%) | 8 (7.3%) | 6 (5.5%) | 4 (3.6%) | 2 (1.8%) | 1 (0.9%) |
| Dyspnoea | 10 (9.1%) | 6 (5.6%) | 4 (3.8%) | 2 (1.9%) | 1 (1%) | 2  (2%) | 8  (7.3%) | 3 (2.7%) | 2 (1.8%) | 2 (1.8%) | 0 | 1 (0.9%) |
| Oropharyngeal irritation* | 8  (7.3%) | 6 (5.6%) | 4 (3.8%) | 2 (1.9%) | 0 | 1  (1%) | 6  (5.5%) | 1 (0.9%) | 1 (1%) | 1 (1%) | 1 (1%) | 0 |
| Headache* | 5  (4.6%) | 2 (1.9%) | 0 | 0 | 2 (2.0%) | 0 | 5  (4.6%) | 1 (0.9%) | 0 | 2 (1.8%) | 0 | 0 |
| Nausea* | 0 | 0 | 0 | 0 | 0 | 0 | 4  (3.6%) | 1 (0.9%) | 0 | 2 (1.8%) | 2 (1.8%) | 1 (0.9%) |

*Progressive decline in AEs
